# Beyond Conventional Pharmacotherapy: Unraveling Mechanisms and Advancing Multi-Target Strategies in Alzheimer’s Disease

**DOI:** 10.3390/ph18121797

**Published:** 2025-11-25

**Authors:** Gabriela-Dumitrita Stanciu

**Affiliations:** Advanced Research and Development Center for Experimental Medicine “Prof. Ostin C. Mungiu”—CEMEX, Grigore T. Popa University of Medicine and Pharmacy Iasi, 700115 Iasi, Romania; gabriela-dumitrita.stanciu@umfiasi.ro

## 1. Pharmacological Management of Alzheimer’s Disease: Current Strategies and Limitations

Alzheimer’s disease (AD) remains the most prevalent form of dementia worldwide, representing a major and escalating global health burden [1]. Despite decades of intensive research and significant advances in understanding its molecular underpinnings, effective pharmacological interventions capable of altering the course of the disease remain elusive [2,3].

Currently approved symptomatic pharmacotherapies, including acetylcholinesterase inhibitors (donepezil, rivastigmine, and galantamine) and the N-methyl-D-aspartate (NMDA) receptor antagonist memantine, provide only modest and temporary relief, primarily targeting cognitive and behavioral manifestations rather than underlying neurodegeneration [4,5]. Acetylcholinesterase inhibitors enhance cholinergic neurotransmission and can transiently improve attention, memory, and global cognitive function, while memantine reduces glutamate-mediated excitotoxicity, offering neuroprotective benefits. Nevertheless, these therapies fail to stop or reverse neuronal loss and their clinical efficacy is often limited to a subset of patients, highlighting the incomplete nature of symptom-focused interventions [4].

In recent years, the development of monoclonal antibodies targeting amyloid-β (Aβ), including aducanumab, lecanemab, donanemab, and gantenerumab, has marked a new era in disease-modifying strategies [6,7]. These compounds aim to enhance the clearance of aggregated Aβ and potentially slow cognitive decline. Clinical trials demonstrate significant reductions in amyloid plaque burden, as evidenced by PET imaging; however, cognitive improvements are modest and inconsistent, and functional gains frequently fail to reach clinically meaningful thresholds [7]. Safety concerns, particularly amyloid-related imaging abnormalities (ARIA) such as cerebral edema and microhemorrhages, in addition to the high cost and limited accessibility of these biologics, have generated ongoing debate regarding their real-world applicability [8,9,10,11].

Beyond amyloid-targeted therapies, several emerging pharmacological strategies are under investigation. Anti-tau agents, including tau aggregation inhibitors and tau-directed antibodies, aim to prevent neurofibrillary tangle formation, a key driver of synaptic loss and cognitive impairment [12]. Approaches targeting neuroinflammation, such as modulators of microglial activation or cytokine signaling, seek to mitigate chronic inflammatory cascades implicated in neuronal injury. Additionally, metabolic modulators, including peroxisome proliferator-activated receptors (PPAR) agonists and insulin-sensitizing agents, are explored for their potential neuroprotective and homeostasis-restoring effects [13]. Early-phase trials of these agents offer promising mechanistic insights, though clinical efficacy remains to be conclusively demonstrated [14].

Clinical experience has also highlighted the heterogeneity of patient responses, with only a subset of individuals deriving measurable benefit from existing therapies. This variability underscores the need for personalized approaches guided by biomarkers, neuroimaging, and genetic profiling. Long-term safety and feasibility concerns, particularly for monoclonal antibodies, necessitate careful patient selection, and ongoing monitoring [8,9,10,11,12,13,14].

Collectively, these observations underscore that single-target interventions are insufficient to address the complex network of pathological events driving AD. Symptom-focused therapies and even amyloid-targeted strategies only partially modulate the underlying molecular, cellular, and systemic disturbances. This landscape highlights the urgent need for multi-target and combination therapies, designed to simultaneously address synaptic dysfunction, protein aggregation, neuroinflammation, and metabolic dysregulation. Such integrative strategies hold the promise of not only ameliorating symptoms but also fundamentally altering the trajectory of neurodegeneration in Alzheimer’s disease.

## 2. Challenges in Translating Molecular Insights into Effective Therapies

Recent advances in molecular neuroscience and systems pharmacology have re-shaped our understanding of AD as a multifactorial and network-driven disorder, rather than a linear amyloid- or tau-centered pathology [15,16]. Neuroinflammation, mitochondrial dysfunction, synaptic failure, oxidative stress, and dysregulated proteostasis interact in a complex cascade that contributes to neuronal loss and cognitive decline [17]. This multidimensional nature of AD suggests that a “one-drug–one-target” approach may be insufficient to address the intricate molecular crosstalk underlying disease progression.

From a mechanistic perspective, several interconnected signaling networks sustain the chronic neurodegenerative process. Mitochondrial dysfunction leads to impaired adenosine triphosphate (ATP) production and excessive generation of reactive oxygen species (ROS), which, in turn, exacerbate oxidative stress and activate redox-sensitive transcription factors such as NF-κB [18]. This activation promotes microglial and astrocytic activation, sustaining a pro-inflammatory milieu that amplifies synaptic damage and neuronal death. Meanwhile, defective proteostasis involving impaired autophagy, ubiquitin–proteasome system dysfunction, and lysosomal stress results in the accumulation of misfolded proteins, including Aβ and hyperphosphorylated tau, which further disrupt intracellular trafficking and mitochondrial dynamics [15,16,17,18].

Synaptic dysfunction represents a convergent point of these molecular changes. Oxidative damage and inflammatory cytokines disrupt glutamatergic transmission and impair long-term potentiation (LTP), while alterations in insulin and lipid signaling pathways affect dendritic spine plasticity [16]. These network-wide disturbances propagate through neuronal circuits, leading to the progressive disconnection of cortical and hippocampal networks that underlie cognitive decline [17].

Translating such multi-layered molecular complexity into therapeutic interventions faces several challenges. Modulating one pathway often triggers compensatory responses in others; for instance, reducing neuroinflammation may inadvertently suppress protective microglial phenotypes, while enhancing autophagy can affect synaptic vesicle turnover or mitochondrial quality control. Moreover, many of these processes are temporally dynamic, what is neuroprotective in early stages (e.g., microglial activation) may become neurotoxic later, making therapeutic timing crucial but difficult to optimize [19]. Additionally, cross-talk between metabolic and inflammatory signaling (e.g., via PPARs, AMP-activated protein kinase—AMPK, and mechanistic target of rapamycin—mTOR pathways) confounds drug design [18]. Compounds such as PPAR agonists can modulate mitochondrial biogenesis, lipid metabolism, and inflammation simultaneously; however, their pleiotropic effects challenge precise dose titration and patient stratification [13]. Effective translation therefore requires a systems pharmacology approach capable of integrating these interdependent molecular pathways rather than isolating single targets.

## 3. Emerging Approaches and Innovative Directions

Emerging concepts such as drug repurposing, multi-target-directed ligands (MTDLs), and precision pharmacology hold substantial promise for redefining the therapeutic landscape [5,20,21,22]. These approaches aim not only to alleviate symptoms but also to modulate multiple pathological pathways simultaneously, potentially restoring homeostatic balance within neuronal networks. Moreover, innovations in gene-editing tools, computational modeling, and artificial intelligence are accelerating the identification of novel targets and the development of mechanism-informed drug candidates.

Drug repurposing, the strategy of identifying new therapeutic uses for existing compounds, has gained renewed interest in AD research. Because many approved drugs already possess well-characterized pharmacokinetic and safety profiles, repositioning can significantly reduce the cost and time of development [23]. Several repurposed agents, such as anti-diabetic PPAR agonists, antihypertensive angiotensin receptor blockers or anti-inflammatory NSAIDs, are being evaluated for their neuroprotective and metabolic effects in AD. Mechanistically, these drugs often act on convergent pathways, such as reducing neuroinflammation, improving mitochondrial bioenergetics or enhancing autophagic clearance of misfolded proteins [23,24].

MTDLs represent another innovative pharmacological concept designed to address the multifactorial nature of AD. Unlike traditional single-target agents, MTDLs combine two or more pharmacophores within one molecule to act on multiple molecular pathways simultaneously: for instance, inhibiting acetylcholinesterase while modulating oxidative stress or metal ion homeostasis [25]. This polypharmacological approach seeks to restore system-level equilibrium and minimize the compensatory mechanisms that often undermine single-target therapies. Rational design of MTDLs is increasingly guided by molecular docking, network pharmacology, and quantitative systems pharmacology models, enabling prediction of synergistic interactions and potential off-target liabilities [26].

Precision pharmacology extends this concept by tailoring interventions to individual molecular profiles. Advances in genomics, transcriptomics, and metabolomics now enable the stratification of patients based on their molecular signatures, genetic risk variants (e.g., APOE ε4), or metabolic phenotypes. Such stratification could identify subpopulations that respond better to specific drug mechanisms, improving efficacy and reducing adverse effects. Integration of multi-omics data with machine learning models further enhances the discovery of predictive biomarkers and drug–target interactions, paving the way for personalized therapeutic strategies [27].

In parallel, gene-editing technologies such as CRISPR/Cas9 and epigenetic modulators are emerging as powerful tools to explore causal mechanisms and potentially correct pathogenic mutations. Although still in preclinical stages, targeted modulation of genes involved in amyloid processing, tau phosphorylation, or microglial activation has demonstrated promising proof-of-concept results [28].

Finally, computational modeling and artificial intelligence (AI) are revolutionizing AD research by enabling the integration of high-dimensional biological data and simulation of disease networks. AI-driven algorithms can predict drug–target affinities, optimize lead compounds, and model disease trajectories, thereby bridging the gap between molecular discovery and clinical translation [29].

## 4. Future Perspectives—From Hope to Implementation

The past decade has seen remarkable progress in understanding the molecular and network-level mechanisms of AD, yet these insights have only partially translated into clinical benefit. The next phase in AD research must focus on transforming conceptual advances into practical, patient-centered interventions, bridging the persistent gap between discovery and delivery.

Future success will depend on the integration of systems biology with clinical translation. By merging data from genomics, proteomics, metabolomics, and neuroimaging, researchers can construct multidimensional models of disease that reveal central signaling pathways and key molecular convergence points—the points at which multiple pathological processes intersect. Targeting these convergence points, rather than isolated molecular entities, could provide more effective leverage over the complex network disruptions driving neurodegeneration. Achieving this vision will require open data infrastructures, cross-disciplinary teams, and strong cooperation between fundamental and clinical research.

The adoption of precision medicine frameworks is equally crucial. Molecular stratification, defining patient subgroups according to shared pathophysiological mechanisms, can guide the rational selection of therapeutic strategies. Adaptive, biomarker-guided clinical trials and AI-assisted patient profiling could dramatically increase the efficiency and success rate of drug development. In parallel, digital biomarkers derived from wearable sensors, speech analysis, or cognitive tracking platforms may enable continuous monitoring of disease dynamics and treatment response in real-world conditions.

Combination and network-based therapies are expected to replace the traditional “one-drug–one-target” paradigm. Coordinated modulation of neuroinflammatory, metabolic and synaptic pathways could help restore functional equilibrium within neural circuits. Computational pharmacology and systems modeling can predict synergistic effects and minimize toxicity, paving the way for personalized multi-modal interventions that blend pharmacological, behavioral, and neurostimulation approaches.

From a technological viewpoint, advances in human-relevant models, such as patient-derived induced pluripotent stem cells (iPSCs), 3D brain organoids, and single-cell multi-omics, are enhancing the fidelity of preclinical testing. These platforms enable mechanistic analysis of disease processes and evaluation of novel gene- and RNA-based therapies with unprecedented precision.

However, true implementation will require ethical, regulatory, and societal alignment. The integration of AI-driven decision tools, data-intensive diagnostics and personalized treatments must be matched by transparent governance, equitable access, and sustained collaboration across academia, industry, and healthcare systems.

In summary, the transition from hope to implementation in AD therapy will depend not only on scientific breakthroughs but also on our ability to interconnect molecular insights, computational intelligence, and clinical reality, ultimately transforming mechanistic understanding into meaningful therapeutic impact.

## Data Availability

The data generated in this study are available from the corresponding author upon reasonable request.

## Abbreviations

The following abbreviations are used in this manuscript:

AD	Alzheimer’s disease
AMPK	AMP-activated protein kinase
ARIA	amyloid-related imaging abnormalities
ATP	adenosine triphosphate
Aβ	amyloid-β
iPSCs	induced pluripotent stem cells
LTP	long-term potentiation
MTDLs	multi-target-directed ligands
mTOR	mechanistic (or mammalian) target of rapamycin
NF-κB	nuclear factor kappa-light-chain-enhancer of activated B cells
NMDA	N-Methyl-D-Aspartate
NSAIDs	non-steroidal anti-inflammatory drugs
PPAR	peroxisome proliferator-activated receptors
ROS	reactive oxygen species
